# Tri-kingdom interactions in bamboo microbiomes: mechanisms of pathogen cooperation and implications for disease management

**DOI:** 10.3389/fmicb.2026.1865230

**Published:** 2026-07-14

**Authors:** Muqadus Zafar, Yingran Wang, Kashf Wajid, Bo Zhao, Ying Cao, Shanglian Hu, Gang Xu

**Affiliations:** 1 College of Life Sciences and Agri-forestry, Southwest University of Science and Technology, Mianyang, China; 2 Engineering Research Center of Biomass Materials, Ministry of Education, Southwest University of Science and Technology, Mianyang, China; 3 Sichuan Provincial Forestry and Grass Land Key Laboratory for Conservation and Sustainable Utilization of Bamboo Genetic Resources in Southwest of China, Mianyang, China

**Keywords:** disease-suppression, management, microbial consortia, microbiome, RNA silencing, synergy coefficient, tri-kingdom synergy

## Abstract

Multi-kingdom disease complexes, where fungi, bacteria, and viruses interact synergistically, are increasingly recognized as a threat to bamboo, a fast-growing Poaceae lineage of high ecological and economic value. However, the mechanisms regulating tri-kingdom disease synergy in bamboo remain poorly understood. This review addresses a central question: Through which molecular and ecological pathways do pathogens from three kingdoms cooperatively enhance bamboo disease severity? We synthesize four key synergy mechanisms: (1) Facilitation of infection: *Fusarium proliferatum* hyphae build physical entry points as well as transport channels that assist *Erwinia* sp. to colonize vascular tissues. (2) Immunosuppression: Bamboo mosaic virus (BaMV; genus *Potexvirus*, family *Alphaflexiviridae*) inhibits host RNA silencing through viral-encoded proteins TGBp1 and CP, which bind small RNA’s and inhibit amplification by RDR6, thereby establishing a permissive environment for secondary invaders, a mechanism inferred from other *Potexvirus* systems, as direct co-infection evidence in bamboo is currently unavailable. (3) Metabolic cross-feeding: fungal virulence enhanced by bacterial metabolites (e.g., lipopeptides, siderophores), although metabolic synergy in bamboo has not been demonstrated. (4) Biofilm protection: scanning electron microscopy reveals bacterial biofilm on the fungal hyphae surfaces that protect pathogens against host defenses. Quantitatively, the *Fusarium-Erwinia* co-infection synergy coefficient of bamboo culm rot is S ≈ 1.8, indicating approximately 80% disease severity. Assuming multiplicative independence among mechanisms, tri-kingdom synergy could exceed S > 3.0, a testable hypothesis. This review identifies the following knowledge gaps: (1) no mycovirus isolated from a bamboo-infecting fungus; (2) no bacteriophage characterized against bamboo bacterial pathogen; (3) no quantified studies involving BaMV; and (4) no genome-wide association studies identifying genetic determinants of synergy. This review proposes that effective biocontrol means disrupting the interfaces of pathogen cooperation - disrupting infection courts, interfering with immunosuppression, chelating iron, and degrading biofilm–rather than introducing beneficial microbes. This review proposes a conceptual framework for cross-kingdom microbial interactions in bamboo-associated microbiomes.

## Graphical abstract

**Figure:**
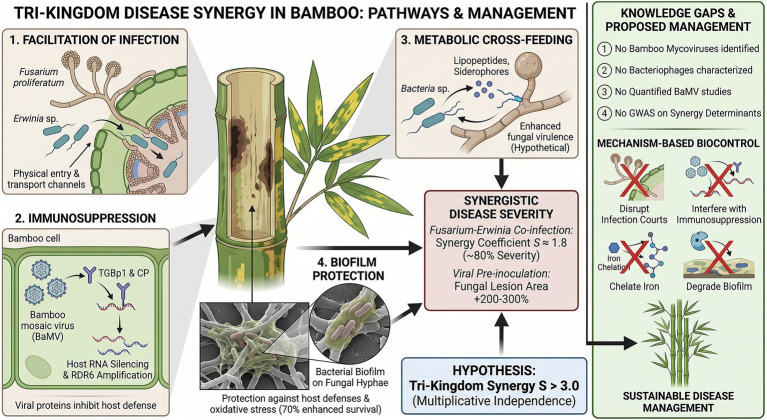
Schematic representation of tri-kingdom interactions within the bamboo microbiome and their collective role in disease suppression and sustainable management.

## 1 Introduction

**Figure 1:**
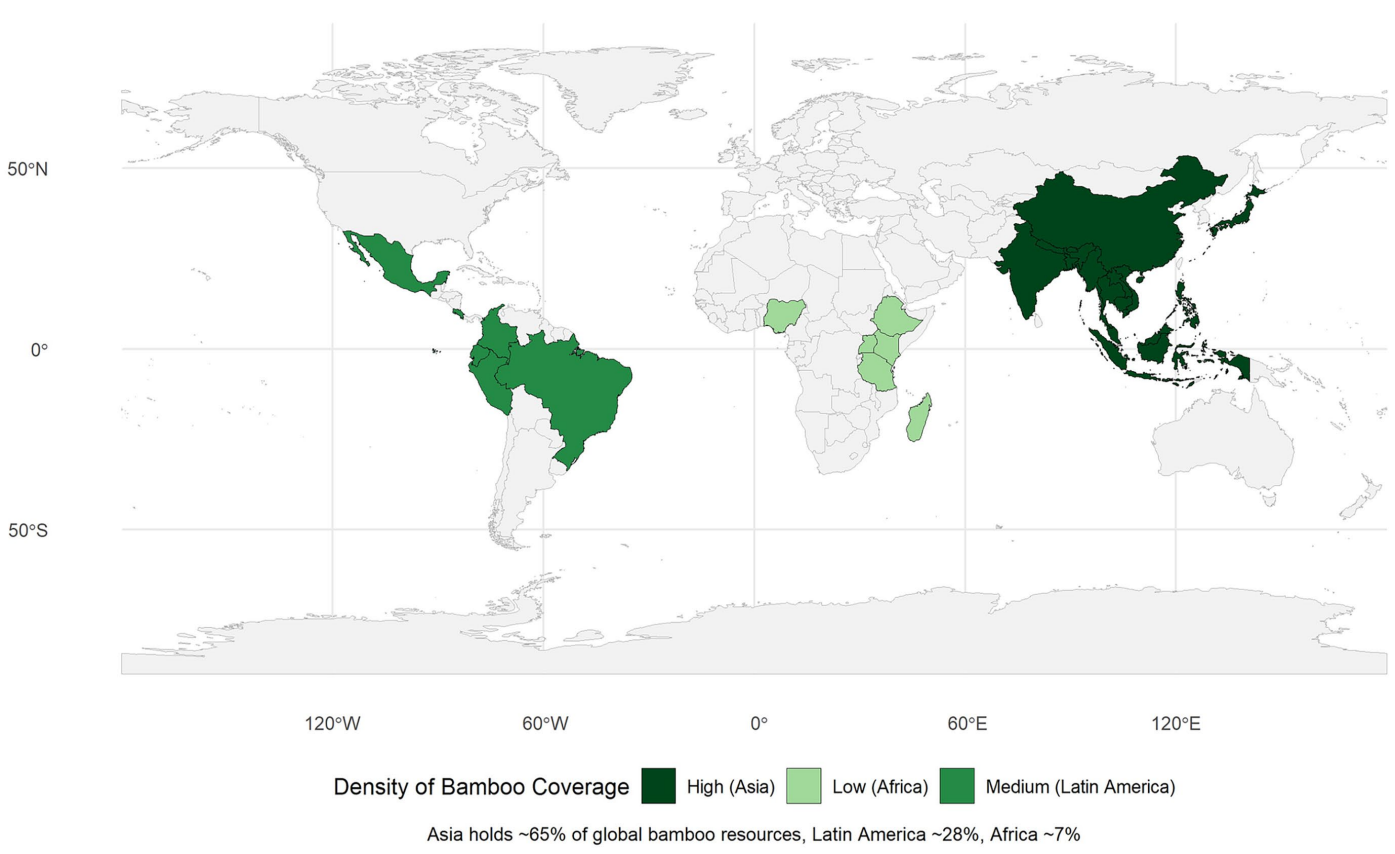
Global distribution and density of bamboo resources. Regions are categorized by relative bamboo density: high in Asia (65% of global resources), medium in Latin America (28%), and low in Africa (7%). The map highlights the concentration of bamboo in tropical and subtropical zones, reflecting its ecological and economic significance in climate-resilient landscapes.

Bamboo, one of the world’s fastest-growing renewable resources, faces escalating threats from complex disease syndromes that jeopardize its ecological and economic potential (Dutta et al., 2025; Sapovadia, 2025). Bamboo is utilized for construction materials, furniture, pulp, paper, food (shoots), and bioenergy, covering an area of approximately ten million hectares worldwide (Hada et al., 2020). It is also a crucial natural resource context for carbon sequestration and ecosystem conservation, as illustrated in Figure 1 (Pan et al., 2023), Bamboo expansion and cultivation can itself reshape surrounding microbial communities (Tian et al., 2020).

**Figure 2:**
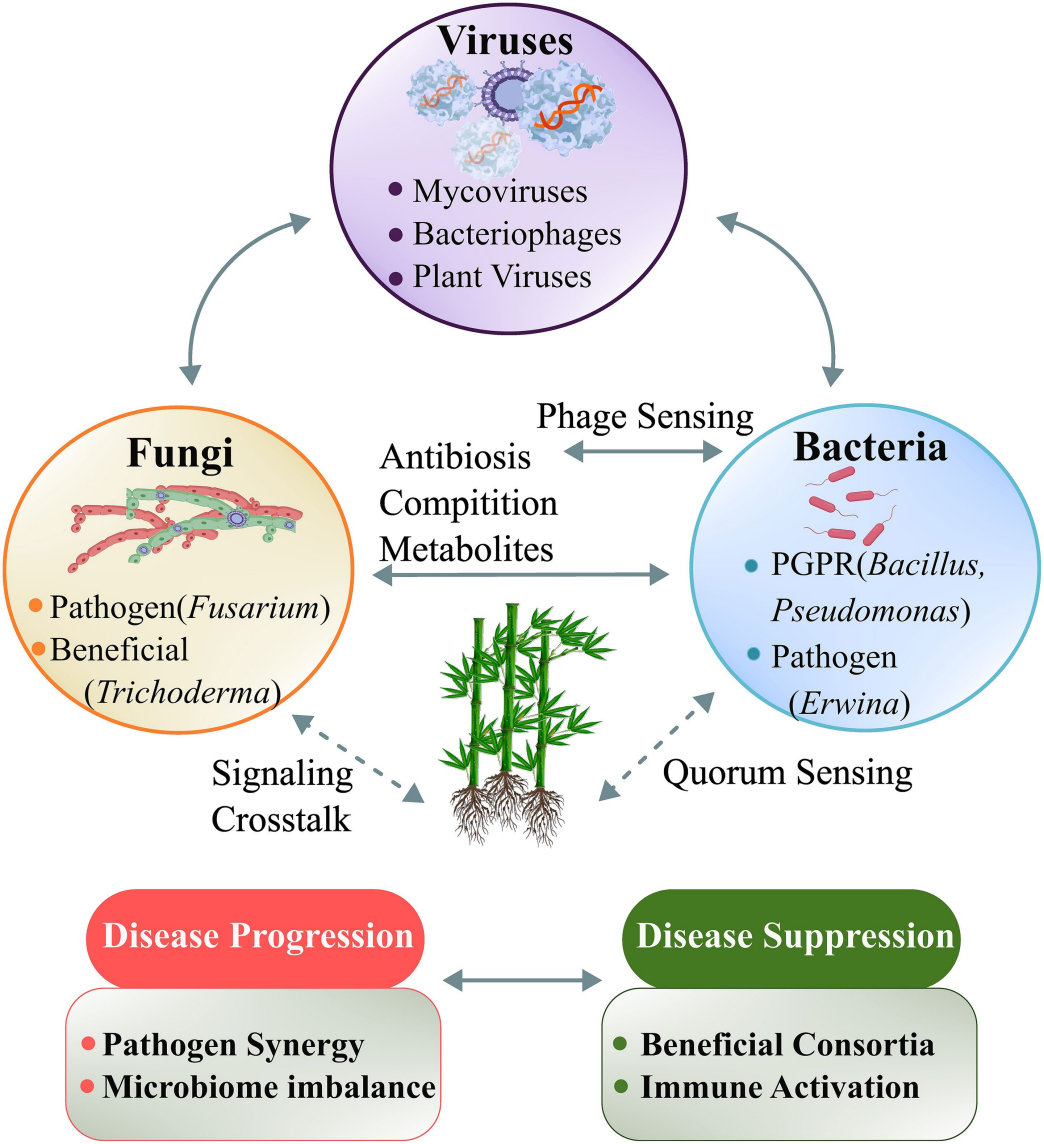
Schematic representation of the bamboo-associated microbiome as a multi-kingdom interaction network. Fungal, bacterial, and viral communities colonizing the rhizosphere, endosphere, and phyllosphere interact through complex ecological and molecular processes, including competition, cooperation, and signaling. These interactions collectively regulate pathogen virulence, microbiome stability, and host immune responses, ultimately determining disease outcomes in bamboo systems.

However, multi-factorial disease syndromes involving synergistic interactions among fungal, bacterial, and viral pathogens are increasingly undermining bamboo productivity causing severe deficits in yield, quality, and ecosystem services (Wang X. et al., 2025; Wang Z. et al., 2025; Wang Y. et al., 2025). As global demands for bamboo increases maintaining plantations, resilience has direct implications for supply chains, rural economies, and carbon management (Hailemariam et al., 2023). Notably, these diseases rarely occur in isolation; co-infections are common, and cross-kingdom synergies exert an effect on the disease severity and on the complexity of their management (Sharma et al., 2026). Major diseases such as leaf blight, culm rot, vascular wilt, and nursery damping-off caused by *Fusarium, Curvularia, Alternaria*, and *Colletotrichum* have led to canopy destruction, weakening of stalks, decreased survival of seedlings, and reduced growth as illustrated in Figure 2 (Thiribhuvanamala et al., 2017). Studies on *Fusarium* infection level in bamboo roots indicate substantial variation with moisture content and farming techniques (Li et al., 2023). *Fusarium* infection leads to severe deterioration of bamboo stalks that are used in paper-making, and consequently reduces carbon fixation (Li et al., 2023).

Chemical control is widely used but is becoming increasingly ineffective. Traditional fungicides provide only temporary control with economic limitations, pathogen resistance, and food safety consequences due to residue accumulation (Maliang et al., 2021). Many fungicides show limited efficacy against bamboo associated pathogens, highlighting the need for more sustainable control strategies. Isolation and omics-based analyses have expanded the identification of antagonists in bamboo microbiomes. These include fungal biocontrol agents, i.e., *Trichoderma, Clonostachys,* and other species like *Gliocladium* sp. These organisms exhibit mycoparasitism activity by producing chitinases, *β*-glucanases, and antifungal compounds such as lytic acid, which lead to the disintegration of the pathogen cell wall (Santra and Banerjee, 2020). Bacterial genera such as *Bacillus, Pseudomonas, and Streptomyces* are widely used as biocontrol agents (Sriragavi et al., 2023). These biocontrol microorganisms do not act in isolation, their effectiveness in disease suppression often depends on cross-kingdom interactions that are mechanistically distinct from pathogenic synergy where pathogens cooperate to enhance disease (Zhang, 2023). Bacterial siderophores have the potential for fungal antagonism, while fungal hyphal networks may facilitate viral transmission. They can synthesize compounds like lipopeptides, phenazines, and siderophores that influence volatile organic compounds (VOCs) (Sriragavi et al., 2023).

Disease synergy occurs when the combined effect of multiple pathogens exceeds the sum of their individual effects (Lamichhane and Venturi, 2015). Formally, the synergy coefficient (S) is defined as:


S=DF+B+V/DF+DB+DV

Here, DF + B + V represents disease severity under tri-kingdom co-infection, while DF, DB, and DV represent disease severity caused by the fungal, bacterial, and viral pathogen individually. A value of *S* = 1 indicates purely additive effects with no synergistic interaction. A value of *S* > 1 indicates synergistic enhancement of disease severity beyond additive expectation, meaning the combined infection causes more disease than the sum of individual infections. A value of *S* < 1 indicates antagonism, where combined infection results in less disease than expected from individual pathogens alone. For example, *S* ≈ 1.8 for *Fusarium-Erwinia* co-infection in bamboo culm rot indicates that combined infection causes 80% more disease than the sum of their individual effects (Li et al., 2023). In model plant systems, S values range from 1.2 representing weak synergy to 4.5 representing strong synergy (Lamichhane and Venturi, 2015). For bamboo, no tri-kingdom S value has been reported. The only quantified pairwise value is *S* ≈ 1.8 for *Fusarium-Erwinia* co-infection in bamboo culm rot (Li et al., 2023). Assuming multiplicative independence among the four synergy mechanisms identified in this review, tri-kingdom synergy could theoretically exceed *S* > 3.0, though this projection remains a testable hypothesis requiring direct experimental validation rather than an established value.

Recent small-scale greenhouse and plot field trials have reported inhibition of damping-off and culm rot when the antagonist isolates were used singly or in consortia (Chauhan, 2021). Viral components remain critically unexplored in bamboo disease ecology. Analyses of plant hosts have revealed extensive viral dark matter on leaves and roots that is even more heterogeneous in both abundance and diversity than previously anticipated (Lin et al., 2023). They may be mycoviruses that induce hypovirulence in plant pathogens, bacteriophages that rearrange the bacterial communities by eliminating and controlling viral targets, or plant viruses that influence signaling pathways and levels of exudates, which in turn affect microbial communities (Siddique, 2020). Viromic studies in bamboo are still in their early stages; model plant studies have demonstrated that mycoviruses can reduce fungal virulence and are transferred by hyphae of target fungi, and phages can provoke direct positive impacts on the control of diseases, promoting the beneficial bacterial community (Huang et al., 2017). The viral entities are viewed as insidious regulators in tri-kingdom microbial niches, having the ability to shift the balance between the pathogenic and benign state by acting on fungal virulence factors, reorganizing bacterial communities, and mediating host immunologic responses.

Viromics and disease ecology in bamboo can now lead to innovative and low-tech methods of control through ecological changes rather than chemical applications (Ahmad et al., 2025). The combination of bamboo-related microbial biocontrol, and viromic discovery, represents a special opportunity for a systems-level interventionist approach, which is ecological on the one hand and precise and scalable on the other (El-Ramady et al., 2023). There is one major challenge that remains: only a substantial part of these studies has concentrated on the individual kingdoms and individual strains; therefore, they have generated helpful information, although not conclusive information. What remains lacking is how fungi, bacteria, and viruses interact in the bamboo ecosystem and how this interaction is harnessed for integrated disease management. Therefore, this specific review summarizes and promotes a tri-kingdom ecological strategy in dealing with bamboo diseases. It not only consolidates much of the important information involving bamboo pathogens and microbial antagonists, but it has also explored viromic space. This review synthesizes advances in tri-kingdom biological control, evaluates the integration of viral elements into biocontrol frameworks, and proposes a holistic framework for sustainable bamboo disease management. The goal is not simply restricted to an amalgamation of literature but enables innovative avenues of dealing with the bamboo diseases by considering mycoviruses, bacterial and viral co-infections with nanotechnologies in diagnosing and delivering these bacteria and viruses. It is important to emphasize that mycoviruses and bacteriophage co-infections in bamboo remain entirely unreported, and no tri-kingdom interaction study has been conducted directly in bamboo. The mechanistic framework proposed in this review is therefore largely extrapolated from model systems and related *Poaceae*, and should be interpreted as a set of working hypotheses rather than established phenomena.

This review synthesizes current knowledge on tri-kingdom interactions in bamboo and addresses the following key objectives: (1) to identify and classify major synergy mechanisms; (2) to quantify synergistic effects where possible; and (3) to highlight knowledge gaps and propose targeted disruption strategies. By integrating evidence from bamboo, related *Poaceae*, and model systems, this work provides a conceptual framework for understanding and managing complex disease interactions in bamboo ecosystems.

An important clarification applies throughout this review: direct experimental evidence for tri-kingdom interactions in bamboo remains extremely limited. No mycovirus or bacteriophage has been isolated or characterized from bamboo, and no quantified tri-kingdom co-infection study has been conducted in this system. Consequently, most mechanistic claims presented here are either inferred from related *Poaceae* systems or extrapolated from model plant studies. To maintain scientific transparency, we consistently distinguish between three evidence levels throughout this manuscript: (1) mechanisms directly demonstrated in bamboo, (2) mechanisms demonstrated in related *Poaceae* or closely related hosts, and (3) mechanisms inferred from model plant systems. Readers are encouraged to interpret all tri-kingdom interaction claims in bamboo as working hypotheses requiring direct experimental validation rather than established biological phenomena.

## 2 Review methodology

This review was conducted through systematic searches of the following electronic databases: Web of Science, Scopus, PubMed, and Google Scholar. The following keywords were used in various combinations: bamboo disease, tri-kingdom interactions, bamboo microbiome, fungal-bacterial-viral synergy, synergy coefficient, Bamboo mosaic virus, *Fusarium* bamboo, bamboo biocontrol, mycoviruses, bacteriophages, bamboo pathobiome, cross-kingdom interactions, plant disease synergy, and microbial consortia. The search covered publications from 1990 to 2025.

Studies were selected based on the following criteria: (1) direct relevance to bamboo disease, microbiome, or biocontrol; (2) relevance to tri-kingdom or cross-kingdom interactions in plant systems, and (3) disease synergy. Studies focusing exclusively on non-plant systems or unrelated hosts were excluded. A total of approximately 80 references were included in the final review.

Given the limited availability of bamboo-specific tri-kingdom studies, evidence from related *Poaceae* and model plant systems was included where relevant and explicitly identified as such throughout the manuscript. The review distinguishes between three evidence levels: (1) mechanisms directly demonstrated in bamboo, (2) mechanisms demonstrated in related *Poaceae* or closely related hosts, and (3) mechanisms inferred from model plant systems. This classification is summarized in Table 1 and discussed in detail in Section 4.6.

**Table 1:** Summary of tri-kingdom pathogen combinations, experimental evidence levels, and confidence scores for proposed synergy mechanisms in bamboo.

**Pathogen combination**	**Host species**	**Evidence level**	**Demonstrated mechanism**	**Confidence score**
*Fusarium + Erwinia*	Bamboo (hybrid)	Direct, quantified	Infection court facilitation (S ≈ 1.8)	Moderate
*Fusarium + Erwinia*	Maize	Experimental	Infection court facilitation	High
BaMV + fungal pathogen	Bamboo	None, hypothetical	Immunosuppression, unconfirmed	Very low
Bean yellow mosaic virus + *Kabatiella caulivora*	Subterranean clover	Experimental	Mixed infection synergy — actual basis for immunosuppression inference (Guerret et al., 2016)	Moderate
*Potexvirus* + fungal pathogen	Model plants	Experimental	Viral immunosuppression	Moderate
*Fusarium* + bacterial metabolites	Maize	Experimental	Metabolic cross-feeding (40–60% toxin increase)	Moderate
*Fusarium* + bacterial metabolites	Bamboo	None, hypothetical	Metabolic cross-feeding, unconfirmed	Very low
*Streptomyces + Fusarium*	Bamboo	Direct	Metabolic antagonism	Moderate
Mycovirus + *Cryphonectria parasitica*	Chestnut	Experimental	Hypovirulence	High
Mycovirus + bamboo fungal pathogen	Bamboo	None, hypothetical	Hypovirulence, unconfirmed	Very low
Bacteriophage + *Erwinia amylovora*	Fire blight system	Experimental	Phage-mediated virulence reduction	Moderate
Bacteriophage + bamboo bacterial pathogen	Bamboo	None, hypothetical	Phage regulation, unconfirmed	Very low
Tri-kingdom (fungal + bacterial + viral)	Bamboo	None, theoretical	S > 3.0 projection	Hypothetical

Confidence scores are defined as follows: High, experimentally demonstrated with quantitative data; Moderate, experimentally demonstrated without quantification or in closely related systems; Very low, hypothetical with no experimental support; Hypothetical, theoretical projection only.

## 3 Bamboo disease complexes: pathogen and synergy potential

Understanding individual bamboo diseases is necessary but not sufficient. Most bamboo pathogens do not occur in isolation. Field observations and molecular diagnostics increasingly reveal that co-infection is the rule, not the exception (MacAulay, 2023; Yu and Wei, 2025) For example, *Fusarium frequently* co-occurs with *Erwinia-*associated shoot rot, and Bamboo mosaic virus (BaMV) precedes with *Colletotrichum* leaf blight. These co-occurrence patterns are based on field observations and molecular diagnostics; the mechanistic basis of these associations has not been experimentally confirmed in bamboo. These co-infection patterns raise a critical question: Do these pathogens simply coexist, or do they actively enhance each other’s virulence? The answer determines whether disease management should target individual pathogens or disrupt synergistic interactions.

Table 2 summarizes the major bamboo pathogens and their potential for cross-kingdom synergy. For each pathogen, we indicate whether pairwise synergy has been reported (in bamboo or related Poaceae) and outline potential mechanisms.

**Table 2:** Major bamboo pathogens and their tri-kingdom synergy potential.

Pathogen	Kingdom	Disease	Documented synergy	Potential synergy mechanism
*Fusarium proliferatum*	Fungus	Wilt, culm rot	Yes (*Erwinia* in maize)	Infection court facilitation
*Curvularia lunata*	Fungus	Leaf blight	Not documented	Immunosuppression via toxin production
*Alternaria alternata*	Fungus	Leaf spot	Yes (BaMV in other plants)	Viral immunosuppression enables fungal colonization
*Colletotrichum gloeosporioides*	Fungus	Anthracnose	Not documented	Unknown
*Erwinia* sp.	Bacterium	Shoot rot, wilt	Yes (*Fusarium* in maize)	Uses fungal hyphae as infection courts
Bamboo mosaic virus (BaMV)	Virus	Mosaic, necrosis	Yes (*Alternaria* in model systems)	Suppresses host RNA silencing

Bamboo diseases often manifest as multi-kingdom complexes resulting from interactions among fungi, bacteria, and viruses (Ghode, 2023). Leaf blights and foliar spots are among most common diseases readily observable in bamboo (Abdel-Rahman et al., 2020). The disease typically presents as necrotic lesions, chlorotic rings, and progressive dehydration of leaves. *Curvularia, Alternaria,* and *Colletotrichum* are the major fungal pathogens affecting bamboo leaves, with *Colletotrichum*-induces anthracnose a significant impact on bamboo leaves and shoots (Elshahawy and Darwesh, 2023; Dey et al., 2023).

In severe outbreaks, foliar disease can significantly reduce the green leaf area and increase susceptibility to secondary infections under humid conditions (Kumar et al., 2021). According to the recent amplicon studies, fungal infections of bamboo are often associated with disrupting bacterial population in bamboo phyllosphere (Wang X. et al., 2025; Wang Z. et al., 2025; Wang Y. et al., 2025). *Fusarium* sp. is commonly associated with vascular wilts in bamboo, progressive yellowing, and death of plant due to disruption of vascular transport (Li et al., 2023). *Fusarium* sp. (*F. oxysporum and F. proliferatum*) derived from soil have been repeatedly reported in wilted bamboo plants and have been associated with the death of seedlings and mature bamboo plants (Li et al., 2023). Co-infection with bacterial pathogens such as *Erwinia* exacerbates *Fusarium* wilt by colonizing vascular tissue already weakened by the fungal pathogen, thereby accelerating disease progression (Thiribhuvanamala et al., 2017).

Comparison of the levels of *Fusarium* across the seasons indicates a significant correlation with outbreaks and lower levels of root nutrients. *Fusarium* sp. along with white-rot or brown-rot group, such as *Poria* sp. and *Schizophyllum* sp. are major contributors to culm rot in bamboo (Li et al., 2023). These pathogens attack bamboo and can render culms unsuitable for industrial applications. Culm rot does not always manifest immediately; when it appears, affected culms are typically mature and have undergone significant decay (Borah, 2006). Researchers who have compiled data regarding mycopathogens associated with bamboo have reported observations of ever-growing wood rot and opportunistic fungi attacking bamboo stalks, particularly because of insect attacks or water rafting incidents (Borah, 2006). Nursery damping-off is a major disease affecting bamboo seedlings, mostly due to association with *Pythium, Rhizoctonia,* or *Fusarium*, significantly limiting plantation expansion and increasing production costs (Thiribhuvanamala et al., 2017). Seedlings can perish in large proportions even in the cases of optimum sanitary conditions and irrigation systems in humid nursery conditions, significantly contributing to the cost and labor involved in the production of healthy seedlings. These nursery issues are among the greatest limitations to formal bamboo plantation (Mehrotra, 1992).

Bacteria and viruses play important roles in bamboo disease systems, acting as primary pathogens or co-pathogens that enhance the fungal disease severity (Lin et al., 2023). The bacterial pathogens and opportunists in bamboo reported are *Erwinia*, which induces shoot wilting and rot diseases, and other bacterial complexes that induce leaf streaks or opportunistically increase decomposition after fungal infection (Joshi et al., 2020). Beyond fungal pathogens, bacterial and viral co-infections strongly influence disease progression. For example, the *Erwinia* sp. can act synergistically with *Fusarium* in causing shoot rot, while Bamboo mosaic virus (BaMV) disrupts host defenses mechanism, increasing susceptibility to fungal pathogens (Cunniffe, 2024). It should be noted that synergistic interactions between *Erwinia* and *Fusarium* have been experimentally demonstrated in maize but not in bamboo, the role of BaMV in increasing susceptibility to fungal pathogens is inferred from other plant virus systems and remains unconfirmed in bamboo pathosystems.

The most well-characterized viral pathogen in bamboo system is Bamboo mosaic virus (BaMV), a member of genus *Potexvirus,* family *Alphaflexiviridae* (Meng and Lee, 2017). However, metaviromic analyses have revealed that bamboo hosts a far more complex viral community than previously recognized. These may involve plant viruses, which may disrupt plant defensive mechanisms, as well as mycoviruses that infect fungal pathogens with which these bamboo species associate and may have a hypovirulent effect on pathogens. To date, no mycovirus has been isolated or characterized from any bamboo-infecting fungus, and the proposed hypovirulent effects remain entirely hypothetical in bamboo systems. *Fusarium* sp. (wilt/culm rot), *Curvularia* sp.*, Alternaria* sp. (leaf blight), *Colletotrichum* sp. (anthracnose/leaf spots), *Pythium* sp. (damping-off), *Ganoderma* sp. and *Schizophyllum* sp. (culm and rhizome rot). Among bambusicolous *Ascomycetes* and *Basidiomycetes* are found, on a regular basis, to be causally predominant under such syndromes (Shukla et al., 2016).

Recent studies have identified new fungal pathogens in bamboo, such as *Neostagonosporella sichuanensis*, *Apiospora* sp., and new species of *Ascomycetes* in several provinces in China (Liu et al., 2024). These new forms seem to be indicative of not only increased detection sensitivity but also actual emerging threats as more area and intensity of plantation is established. BaMV is currently the most studied viral pathogen in bamboo and serves as a model system for studying host-virus interactions (Cheng, 2017). However, it should be noted that BaMV research has focused primarily on molecular virology and host factor interactions; its role in tri-kingdom disease synergy with fungal or bacterial co-pathogens in bamboo has not been experimentally investigated. Simultaneously, phyllosphere-related viral communities by means of shotgun viromic analysis have yielded a tremendous diversity of viruses previously unrecognized to be implicated in bamboo disease (Siddique, 2020). The fact that large quantities of unknown viral sequences were identified in the leaves and the rhizosphere samples is a case that supports the use of viromic screens. However, the functional roles of these viral sequences in bamboo disease remain entirely unknown, and their contribution to tri-kingdom pathogen interactions is currently speculative. Recent publications demonstrate the dynamic nature of the communities of bamboo pathogens and the impact of environmental drivers (Zhang et al., 2025). Fungal seasonal sampling in naturalized bamboo has been highly diverse in terms of density of pathogen and community structure in regimes of moisture and temperature. Overall, such infected roots and soil contain lower levels of nutrients compared to clean areas.

Ongoing surveillance across Asia continues to identify both emerging and established fungal pathogens in the bamboo system (Geng et al., 2020). Such disease burdens may have a significant economic and ecological effect. Smallholders and industries that depend on the quality of bamboo lose a lot of money when diseases affect nurseries and plantations (Maina, 2023). However, the disease ecology may potentially be influenced by emerging pathogen species, expanding viromes, and opportunistic bacterial species, though the mechanistic basis of these interactions remains poorly characterized (Zhang et al., 2025). The general implication because of culm rot disease, nursery losses, and generalized large-scale virome would therefore necessitate methods beyond chemical control as a measure to overall baseline functioning. Based on the evaluation of various recent publications on the ecology of bamboo disease, it turns out that it incorporates a variety of methods of ecological functioning in general. Several pathogens, including *Fusarium*, *Curvularia*, *Alternaria*, *Colletotrichum*, and wood rot fungi, increase their adverse effects on aggregate stocks of bamboo that operate (Kawadkar, 2012).

**Figure 3:**
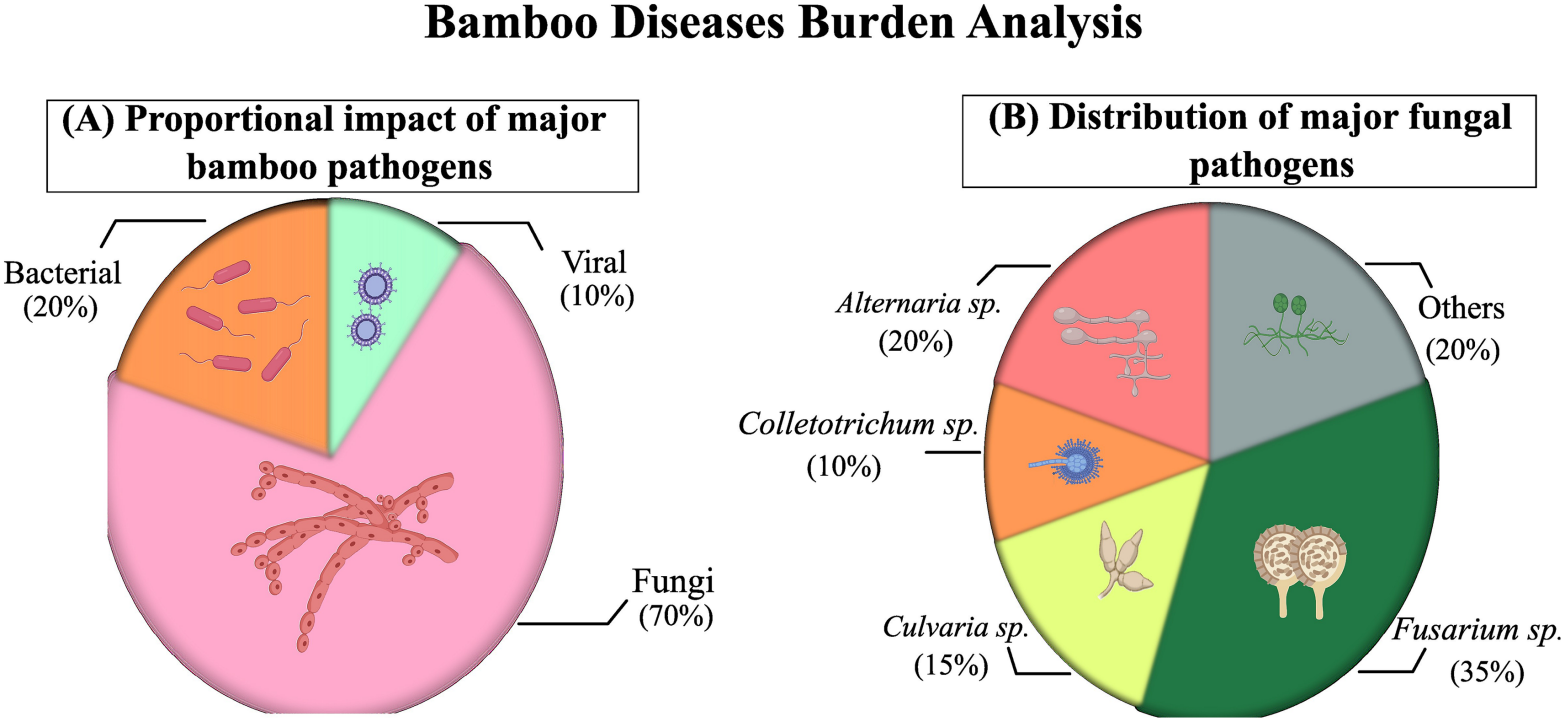
Burden of major bamboo diseases by pathogen type and distribution of dominant fungal genera. **(A)** Proportional contribution of major pathogen groups affecting bamboo, showing fungi as the predominant agents (70%), followed by bacteria (20%) and viruses (10%). **(B)** Relative distribution of key fungal pathogens, with *Fusarium* sp. representing the largest share (35%), followed by other fungi (20%), *Curvularia* sp. (15%), *Colletotrichum* sp. (10%), and additional minor fungal groups (20%). Together, these data highlight the dominant role of fungal pathogens in bamboo disease epidemiology and the diversity within fungal communities.

In summary, the bamboo pathobiome consists of diverse fungi, bacteria, and viruses that frequently co-infect. These interactions form complex disease networks that influence disease severity and progression. Section 4 examines mechanistic evidence for true synergistic interactions (Figure 3).

## 4 Mechanisms of tri-kingdom disease synergy in bamboo

**Figure 4:**
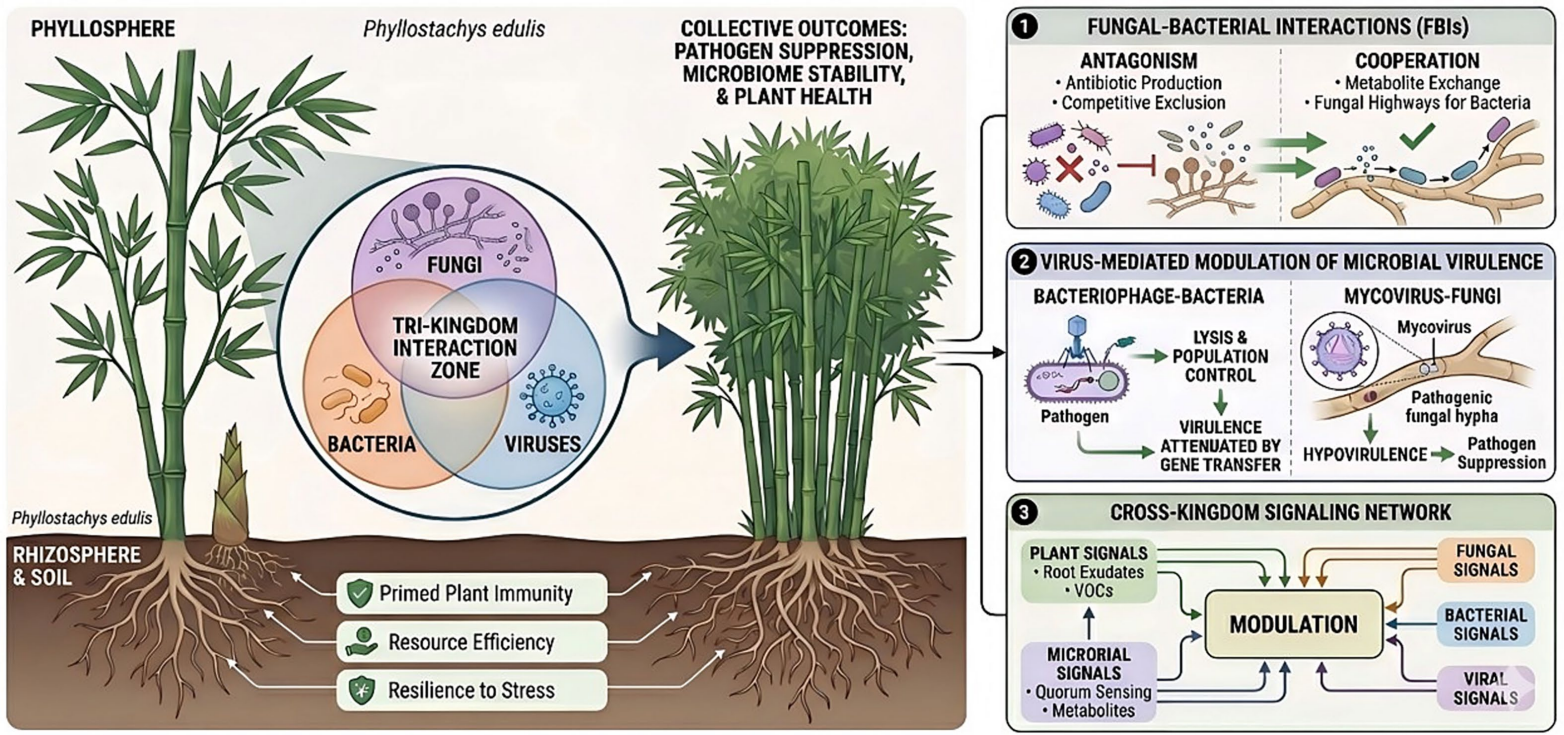
Conceptual representation of tri-kingdom interactions among fungi, bacteria, and viruses in bamboo-associated microbiomes. The figure illustrates direct and indirect interactions, including fungal-bacterial antagonism and cooperation, virus-mediated modulation of microbial virulence, and cross-kingdom signaling that collectively influence pathogen suppression, microbiome stability, and plant health.

Having established that bamboo pathogens frequently co-infect, this section addresses the central question: through what mechanisms do fungi, bacteria, and viruses cooperatively enhance disease severity? We identify four distinct synergy mechanisms, each supported by evidence from bamboo or from related Poaceae and model systems. Figure 4 provides a conceptual overview of these mechanisms, which are detailed in Sections 4.1–4.4.

### 4.1 Mechanism 1: infection court facilitation

Infection court facilitation occurs when one pathogen creates physical entry points or transport pathways for another pathogen, thereby increasing disease severity beyond what either pathogen achieves alone. The interaction between fungal and bacterial microbiota in bamboo plant tissues and the surrounding soils play a central role in the disease dynamics, influencing both pathogens virulence and host resistance (Zhang et al., 2019). Bamboo-associated endophytic fungi, including *Fusarium*, *Arthrinium,* and *Xylaria,* are widespread and exhibit diverse ecological roles ranging from symbiosis to pathogenicity in leaves, culm, and roots (Hyde et al., 2002). Simultaneously, endophytic bacterial strains of moso bamboo (*Phyllostachys edulis*) exhibit plant growth-promoting traits including nutrient solubilization and antimicrobial activity against phytopathogens (Yuan et al., 2015).

Evidence from related Poaceae and model plant systems suggests that fungal-bacterial interactions may be antagonistic, where competing microorganisms reduce pathogen populations through antimicrobial compound secretion, though direct mechanistic evidence in bamboo remains limited (Wang X. et al., 2025; Wang Z. et al., 2025; Wang Y. et al., 2025). For example, *Streptomyces bambusae,* identified in the bamboo rhizosphere, exhibits both antifungal and antibacterial properties, representing a clear example of native bacterial populations suppressing fungal pathogens (Sriragavi et al., 2023). In contrast, synergistic interactions may occur; bacterial metabolites can induce fungus secondary metabolism, which antagonize pathogens, while fungal hyphal networks may facilitate bacterial dispersal into plant tissues (Li et al., 2023). The synergistic interactions between fungal and bacterial pathogens have not been directly demonstrated in bamboo and are inferred from studies in other plant systems. Positive results have been seen with beneficial fungus-bacterium consortia in other plant systems with respect to disease suppression and growth promotion (Wu et al., 2023). Applied to bamboo, these findings suggest that synthetic microbial consortia could suppress soil-borne pathogens such as *Fusarium proliferatum*, the pathogen causing the basal rot, and enhancing the root development and plant vigor. As noted in the Introduction, the only quantified bamboo synergy value remains S ≈ 1.8 for this pathogen pair.

### 4.2 Mechanism 2: immunosuppression

Immunosuppression occurs when one pathogen, typically a virus, suppresses host defense responses, creating a permissive environment for fungal and bacterial invasion (Fishman, 2013). The previously unexplored but potentially important part of disease ecology in bamboo is viruses that infect fungi (mycoviruses). Mycoviruses can induce hypovirulence in plant pathogenic fungi by interfering with fungal genes expression and virulence pathways, as demonstrated in biological control of chestnut blight (Mang et al., 2025). The absence of characterized mycoviruses in bamboo is discussed in Section 5.

A well-characterized plant virus in bamboo is Bamboo mosaic virus (BaMV), a member of the genus *Potexvirus*, family *Alphaflexiviridae*, which infects a wide range of bamboo species (Wu et al., 2025). Typical symptoms of the BaMV infection include chlorotic mosaic and necrosis of foliage resulting in yield loss and reduces shoot quality (Huang et al., 2022). In model systems, BaMV infection prior to fungal inoculation increases lesion size by 200–300% compared to the fungus alone, demonstrating immunosuppression-driven synergy (Guerret et al., 2016). BaMV is a plant pathogen, rather than a mycovirus; however, its interactions with other microbial communities likely determine disease outcomes. For example, virus-mediated changes in host defense signaling and phyllosphere exudates can alter bacterial and fungal community structures, potentially creating niches of pathogenic opportunists or beneficial microbial antagonists (Bi et al., 2025).

### 4.3 Mechanism 3: metabolic cross feeding

Metabolic cross-feeding occurs when metabolites produced by one pathogen enhance the growth, virulence, or survival of another (Ramsey et al., 2011). Chemical signaling mediated by diffusible metabolites, including quorum-sensing molecules, secondary metabolites, and volatile organic compounds, is one of the major mechanisms underlying cross-kingdom interactions (Ren et al., 2024). These signaling molecules regulate microbial behavior by conveying information about population density, nutrition availability, and environmental conditions, thereby regulating microbial growth, antagonism, and cooperation (Rice et al., 1999).

Bacterial quorum-sensing signals have been observed to impact fungal development and virulence, while fungal-derived metabolites influence bacterial motility, biofilm formation, and antimicrobial production, collectively contributing to suppression of pathogens (Wongsuk et al., 2016). Direct evidence for quorum sensing-mediated cross-kingdom interactions in bamboo microbiomes remains to be established, as demonstrated in other crop systems. Although direct experimental evidence in bamboo is limited, metagenomic and metabolomics studies of bamboo-associated microbiomes show that genes involved in signal production and perception are widespread. This suggests that similar communication frameworks may operate in bamboo microbiomes.

Another important but overlooked layer of regulation in microbial interaction networks involves viruses. Mycoviruses can modify fungal physiology and pathogenicity by altering gene expression, RNA silencing pathways, and cellular metabolism, often leading to hypovirulence (García-Pedrajas et al., 2019). Recent studies from other plant systems show that pathogenic fungi infected by mycoviruses exhibit reduced virulence and diminished capacity to colonize host tissues (Nuss, 2005). In contrast, bacteriophages regulate bacterial populations through host-specific infection, lysis, and horizontal gene transfer, thereby preventing the dominance of specific bacterial taxa and maintaining community balance (Alkhalil, 2023). Although mycoviruses and bacteriophages in bamboo ecosystems remain uncharacterized, and no functional characterization of viral agents in bamboo has been reported, recent viromic surveys indicate substantial viral diversity, suggesting that viral agents play an influential role in microbial balance and disease diversity (Bhardwaj et al., 2017).

Metabolic interaction is another key mechanism that contributes to the stability and functionality of tri-kingdom microbial consortia (Berrios et al., 2023). Competition and cooperation among microorganisms often occur for essential resources such as iron, carbon, and nitrogen, leading to niche differentiation and metabolic complementation. Beneficial bacteria can inhibit the fungal pathogens either by means of the siderophore-mediated iron sequestration or antibiotic synthesis, and the fungal hyphae can redistribute the nutrients and disperse microbes in plant ecosystems (Dhuldhaj and Pandya, 2021). Similar perennial and crop systems indicate that such coordinated interactions increase disease resistance and ecosystem stability, especially under dynamic environmental conditions.

On a larger scale, co-occurrence network analyses have shown complex bacteriophage interactions determining microbiome dynamics that can be generalized to bamboo, wherein libraries structured networks can be utilized to confront bacterial pathogen outbreaks and maintain the beneficial microbial processes (Zhang J. et al., 2024; Zhang M. et al., 2024). A critical distinction should be emphasized: metabolic antagonism (one metabolite suppressing another kingdom) is well documented in bamboo (e.g., *Streptomyces* antifungals), (Sriragavi et al., 2023) but true metabolic synergy where bacterial metabolites enhance fungal virulence has been demonstrated in maize (40–60% increase in toxin production) but not yet in bamboo (Wu et al., 2017).

### 4.4 Molecular and host-mediated regulation of tri-kingdom synergy

Beyond the four synergy mechanisms, additional layers of regulation involve chemical signaling, host immunity, and viral modulation. Biofilm-structured microbial communities embedded in an extracellular matrix can physically protect pathogens from host defenses in other plant systems (Arasu, 2025); however, this mechanism has not been investigated in bamboo pathosystems. Most molecular regulation occurs through diffusible signals rather than physical barriers alone. Phage-bacterial network in bamboo have not yet been characterized, but analyses in other vegetative microbiomes have demonstrated that phages have the potential to reduce the presence of harmful microbes and at the same time increase the population of useful taxa, resulting in enhanced disease and plant resistance (Wang et al., 2024). Bacteriophages against bacterial wilt pathogens, e.g., *Erwinia* sp., in the bamboo scenario may reduce virulence, prevent systemic colonization, and enhance resistance to co-infection with fungal pathogens (Ke et al., 2024). The proposed role of bacteriophages in bamboo system is entirely hypothetical, as no bacteriophage targeting a bamboo bacterial pathogen has been isolated or characterized yet.

The current mechanistic understandings are based on model plants and agricultural systems; most of these processes are evolutionary conserved and likely to operate within bamboo ecosystems (Dutta et al., 2025). The reinforcement of the mechanistic structure of tri-kingdom interactions is key to the translation of ecological observations into effective and long-term disease management plans.

Beyond direct microbe interactions, the plant host actively regulates tri-kingdom dynamics through immune signaling, root exudates, and secondary metabolites. Host-mediated selection can favor the beneficial microorganisms, but proliferation of pathogens may be limited, thereby indirectly shaping the cross-kingdom interactions (Jones et al., 2019). Beneficial microbes can activate plant defense pathways, creating a feedback loop in which host immunity is reinforced through cooperative interactions with mutualistic microorganisms (Nishad et al., 2020). Despite the lack of mechanistic research on bamboo, increasing evidence indicates that plant physiological and environmental conditions strongly influence microbial assembly and functions, which motivates a stronger encouragement towards incorporating host regulation in models of tri-kingdom interaction.

**Figure 5:**
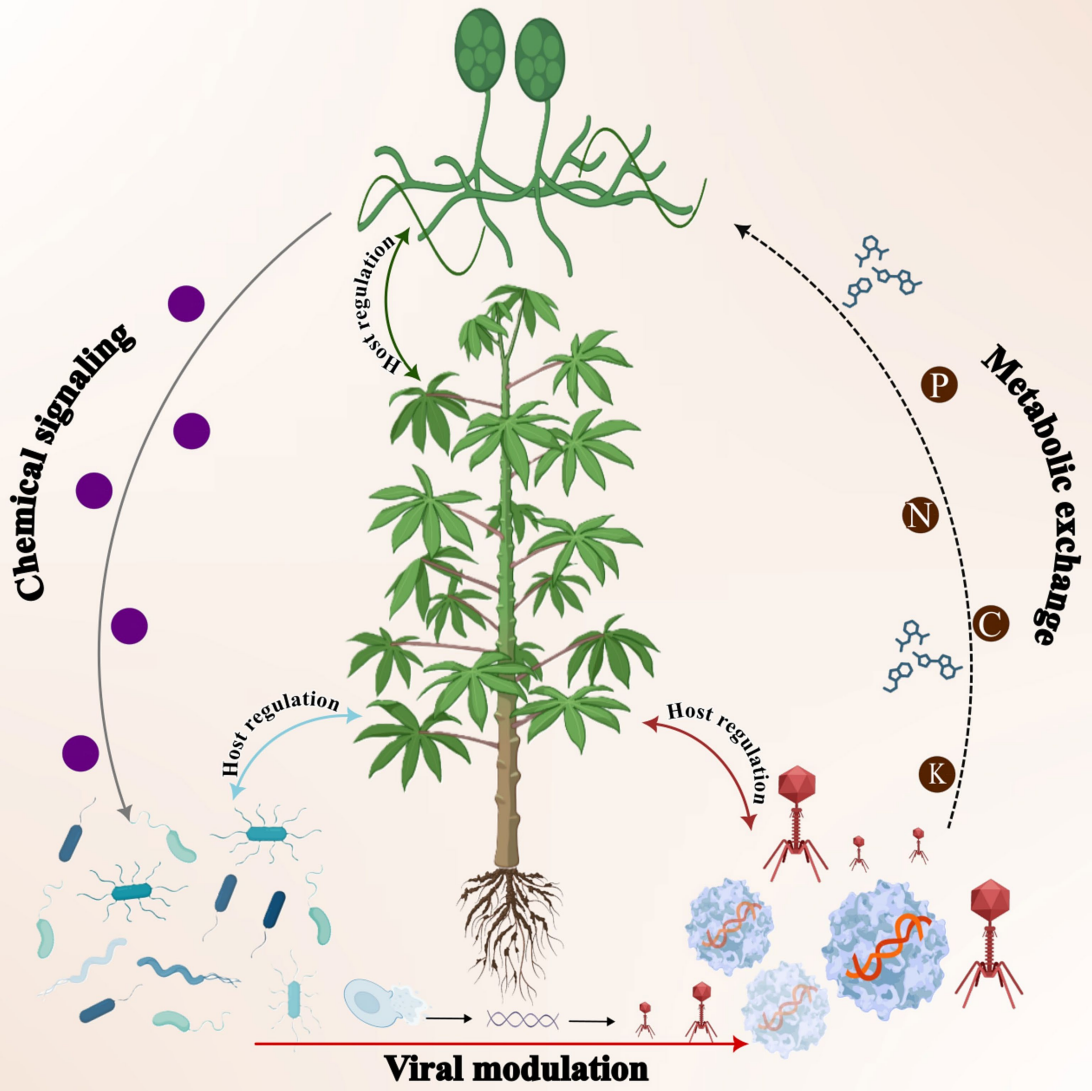
Conceptual model of molecular mechanisms governing tri-kingdom interactions among bacteria, fungi, and viruses in plant-associated microbiomes, integrating chemical signaling, viral modulation, metabolic exchange, and host-mediated regulation.

Molecular evidence indicates that tri-kingdom interactions are governed by coordinated signaling, viral modulation, metabolic exchange, and host-mediated regulations rather than pairwise interactions (Berrios et al., 2023). Integrating mechanistic knowledge from bamboo and other plant systems forms the conceptual framework for how tri-kingdom interactions act as a contributor to pathogen suppression, microbiome stability, and plant health. Figure 5 integrates the three major molecular regulatory layers: chemical signaling, host recognition, and viral modulation that govern tri-kingdom interactions in bamboo.

### 4.5 Integrated tri-kingdom networks and plant immune modulation

#### 4.5.1 Pathogenic synergy versus beneficial consortia: a conceptual distinction

A fundamental conceptual distinction must be maintained throughout this review. Pathogenic tri-kingdom synergy refers to cooperative interactions among fungal, bacterial, and viral pathogens that collectively enhance disease severity beyond what individual pathogens achieve alone. In contrast, beneficial tri-kingdom consortia refer to cooperative interactions among antagonistic microorganisms, including biocontrol fungi, bacteria, and viral agents, that collectively suppress disease. These two phenomena are functionally opposite: pathogenic synergy drives disease development while beneficial consortia drive disease suppression. Although both involve cross-kingdom microbial cooperation, their ecological roles, mechanistic bases, and management implications are entirely distinct. Sections 4.1–4.4 address pathogenic synergy mechanisms exclusively, while Section 5 addresses how beneficial consortia can be designed to disrupt these mechanisms.

#### 4.5.2 Tri-kingdom network dynamics and host immune modulation

The association of fungi, bacteria, and viruses creates complex interaction networks with implications for both disease development and disease suppression. It is important to distinguish between pathogenic tri-kingdom synergy, where pathogens cooperate to enhance disease severity, and beneficial tri-kingdom interactions, where antagonistic microorganisms cooperate to suppress disease. These two phenomena are mechanistically related but functionally opposite (Dey et al., 2023). Synthetic microbial communities (SynComs) composed on beneficial fungi (*Trichoderma* sp.), bacteria (*Bacillus* sp. *and Streptomyces* sp.), and possibly viral organisms have been demonstrated to cause enhancement of rhizosphere colonization, and enhance soil enzymatic activity as well as priming plant immune responses in bamboo, resulting in growth promotion and disease suppression (Lin et al., 2023). While fungal-bacterial SynComs have been observed in bamboo rhizosphere studies, the viral component of such consortia remains entirely hypothetical, as no viral biocontrol agent has been tested in bamboo to date.

The combined tri-kingdom networks work via coordinated regulation of signaling networks, metabolic interactions, and restructuring of microbial communities (Guo et al., 2024). Many microbes can create systemic acquired resistance (SAR) and induced systemic resistance (ISR) in the host, as well as improve defense gene expansion and strengthen cell wall defenses to pathogen invasion (Dey et al., 2023). These processes may also be acted upon by viral elements such as mycoviruses or plant viruses, which modify defense pathways and are acquired from changes in defense against them or reinforce defense depending on combinations between viral interactions and particular host responses (Zhu et al., 2022).

Rhizosphere and endophytic microbiome studies in bamboo indicate that both the bacterial and fungal communities have dynamics and are sensitive to environmental factors and host factors (Li et al., 2023). Organic supplements can have profound effects on the composition of root-associated bacteria that in turn can mediate impacts on how the microbiome, fungal, and viral respond to disease susceptibility or resistance.

### 4.6 Evidence levels: demonstrated vs. hypothetical mechanisms

A critical distinction must be maintained when interpreting the mechanistic evidence presented in this review. Given the limited experimental work conducted directly in bamboo systems, the four tri-kingdom synergy mechanisms described in Sections 3.1–3.4 vary substantially in their evidentiary support.

The only mechanism with direct quantitative support in bamboo is infection court facilitation, for which a pairwise synergy coefficient of S ≈ 1.8 has been documented for *Fusarium-Erwinia* co-infection in bamboo culm rot (Li et al., 2023). All other mechanisms, including immunosuppression, metabolic cross-feeding, and biofilm protection, are inferred from related *Poaceae* or model plant systems and remain unexplored in bamboo.

Specifically: (i) BaMV-mediated immunosuppression is mechanistically plausible based on known *Potexvirus* biology, but no co-infection study involving BaMV and a fungal or bacterial pathogen has been conducted in bamboo, (ii) metabolic cross-feeding has been demonstrated in maize but not in bamboo, (iii) biofilm-mediated protection has been characterized in other polymicrobial systems but not in bamboo pathosystems, and (iv) no mycovirus or bacteriophage has been isolated or characterized from bamboo.

The proposed tri-kingdom synergy coefficient of S > 3.0 is a theoretical projection based on multiplicative independence of pairwise interactions and should be treated as a testable hypothesis rather than an experimentally validated value. Future research priorities should focus on direct experimental validation of these mechanisms in bamboo systems.

Table 1 provides a comprehensive summary of pathogen combinations discussed in this review, their host systems, experimental evidence levels, demonstrated mechanisms, and associated confidence scores, clearly distinguishing bamboo-specific evidence from extrapolations based on other systems.

## 5 Disrupting tri-kingdom synergy: strategies for sustainable biocontrol

**Figure 6:**
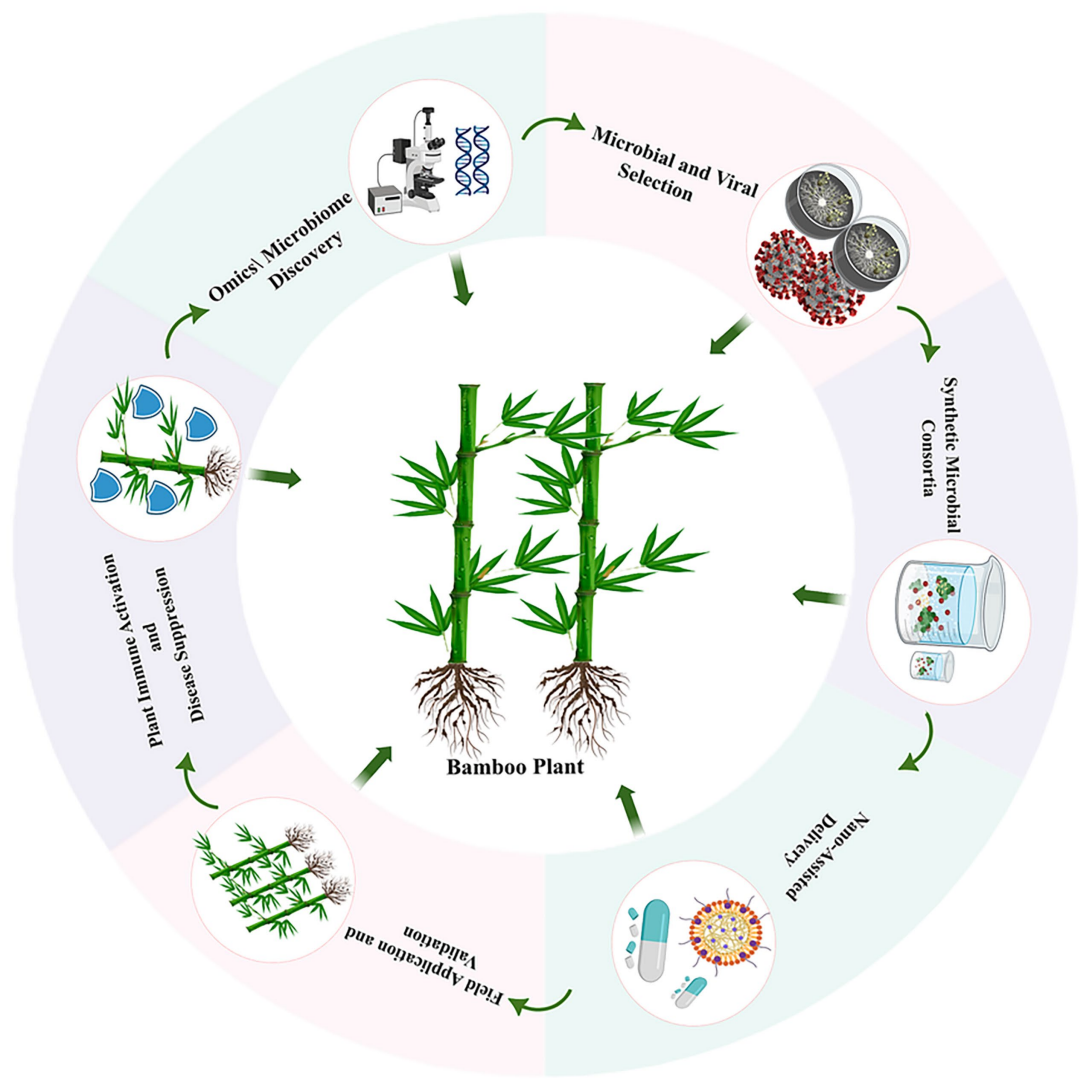
Framework for the application of tri-kingdom biocontrol strategies in sustainable bamboo disease management. The schematic highlights the integration of fungal, bacterial, and viral components with host plant responses, environmental factors, and management practices to enhance disease suppression, microbiome stability.

Section 4 outlines four synergy mechanisms that define specific intervention points. Rather than simply introducing beneficial microbes, effective biocontrol strategies should target the interaction interfaces through which pathogens synergy operates. It is important to clarify that the beneficial tri-kingdom interaction discussed in this section, involving antagonistic bacteria, fungi, and viral agents, is not mechanistically analogous to pathogenic synergy. Rather, they represent disruption of pathogenic cooperation through ecological manipulation of the microbiome. Each synergy mechanism is mapped to the corresponding disruption strategy below. In the absence of direct evidence in bamboo, we rely on model systems and propose testable hypotheses. In comparison to the traditional methods, tri-kingdom-based interventions consider the ecological fact of bamboo systems, where disease manifestations are determined by the interaction of coexisting microbial networks and host–microbe interactions. It is therefore essential to translate mechanistic understanding into practical applications to gain sustainable disease suppression and improved bamboo plant resilience. Figure 6 provides a conceptual framework for translating tri-kingdom synergy mechanisms into practical biocontrol strategies.

### 5.1 Disrupting infection court facilitation: synthetic consortia

The previously described antagonist consortia, including *Trichoderma, Clonostachys, Gliocladium, Bacillus, Pseudomonas,* and *Streptomyces,* are key components of healthy bamboo rhizosphere and endophytic communities (Wang X. et al., 2025; Wang Z. et al., 2025; Wang Y. et al., 2025). Infection court facilitation can be disrupted by preventing fungal hyphal adhesion or by introducing bacteria that outcompete pathogens for accessing hyphal networks. The antagonist consortia target pathogenic fungi through mycoparasitism, antibiosis, nutrient competition, and niche exclusion (Wang X. et al., 2025; Wang Z. et al., 2025; Wang Y. et al., 2025).

The use of fungal and bacterial antagonists in synthetic consortia is more effective than single-strain inoculants, because it promotes functionality and ecological stability. Experiments in nurseries and greenhouse trials of other plants suggest that co-inoculation using fungal and bacterial biocontrol agents enhances the reduction in damping-off and soil-borne diseases and promotes root growth and nutrient acquisition (Chauhan, 2021). Similar results have been reported across multiple crop systems, where multi-strain consortia outperform individual agents under variable environmental conditions (Niu et al., 2020).

### 5.2 Targeting immunosuppression: viral agents as future tools

Mycoviruses represent one of the most promising tools for targeting immunosuppression, including hypovirulence in pathogenic fungi and have been successfully used on other plant systems to reduce disease severity without disrupting ecosystem balance (Niu et al., 2020). Despite very few direct applications of mycoviruses to bamboo, viromic surveys have indicated substantial mycoviruses diversity in bamboo-associated microbiome is very high, thereby indicating that natural mycoviruses can have already impacted fungal pathogenicity in these systems (Xie and Jiang, 2024).

Simultaneously, bacteriophages targeting bacterial pathogens such as *Erwinia* represents a precise strategy for suppressing bacterial co-pathogen, thereby indirectly reducing fungal disease severity (Ke et al., 2024). Phages may also aid bacterial community balance by controlling the bacterial population dynamics and preventing dysbiosis and helping stabilize an effective population of beneficial bacteria.

### 5.3 Disrupting metabolic cross-feeding and biofilm protection

Metabolic cross-feeding and biofilm protection can be disrupted by interfering with quorum sensing, chelating iron, or degrading biofilm matrices. At the molecular level, tri-kingdom interactions are regulated by some complex signaling networks, metabolic interactions, and gene regulatory processes. Quorum-sensing molecules produced by *Bacillus* and *Pseudomonas*, including iturins, fengycins, and phenazines, disrupt pathogen cell membranes and interfere with fungal respiration (Zhang J. et al., 2024; Zhang M. et al., 2024). Such metabolites destabilize the cell membrane of the pathogens and disrupt fungal respiration, making them less fit (Ning et al., 2024).

Fungal antagonists such as *Trichoderma* sp. directly degrade pathogen cell wall by secreting cell wall-degrading enzymes, including chitinases, *β*-glucanases, and proteases (Hodiyah et al., 2024). Expression of these enzymes is tightly regulated by bacterial-derived signaling compounds and nutrient availability, providing evidence of cross-kingdom gene regulation. Transcriptomics studies of bamboo and other perennial crops show that co-cultivation of beneficial fungi and bacteria shows the upregulation of genes linked to mycoparasitism, stress resistance, and secondary metabolism, thereby increasing disease suppression (Zhao et al., 2025).

Another regulatory layer of the tri-kingdom interactions is made up of host-mediated molecular responses. The beneficial microbes stimulate bamboo defense signaling pathways, such as jasmonic acid, salicylic acid, and ethylene-mediated networks, which cause induced systemic resistance (Wang et al., 2021). This is coupled with upregulation of defense-related genes, pathogenesis-related protein, lignin biosynthesis enzymes, and reactive oxygen species-scavenging system (Tayi, 2025). The interplay between microbial antagonism and host immune priming forms a multi-layered defense system that restricts the pathogen’s development and prevents disease.

Collectively, these molecular interactions indicate that tri-kingdom disease synergy in bamboo is not due to a single antimicrobial action but instead coordinated network of signaling, metabolic regulation, and host-microbe communication.

### 5.4 Field validation, and climate resilience

The success of tri-kingdom biocontrol measures must be validated through field experiments beyond controlled trials. Microbial ecology is strongly influenced by the soil properties, environmental heterogeneity, microbial diversity, and bamboo growth stage. Field studies in bamboo systems demonstrate that biocontrol performance depends on site-specific soil physicochemical properties, moisture content, and plantation age, which highlights the need to optimize the processes of specific sites.

Long-term field-testing duration is necessary to determine disease suppression efficacy, microbial persistence, and effects on bamboo growth and yield. Such data is essential when it comes to improving the strategy of application and hence making tri-kingdom interventions sustainable.

### 5.5 Biosafety, regulatory, and ecological considerations

The use of multi-kingdom biocontrol agents, particularly viral components, requires careful consideration of biosafety and regulatory concerns (Fadiji et al., 2025). Mycoviruses are generally non-pathogenic and host-specific, posing no harm to animals or plants; however, their ecological behavior in the bamboo ecosystem requires further evaluation of their environmental persistence and interactions (Aoki et al., 2009). Similarly, the use of bacteriophage application should be carefully designed to prevent the harmful impact on beneficial bacteria in the rhizosphere and endosphere.

Regulatory systems supporting viral biocontrol agents continue to evolve, especially within the forestry and perennial crop systems (Wagemans et al., 2022). As bamboo is a sustainable energy and climate-smart resource, tri-kingdom biocontrol strategies in this case must be in line with environmental protection standards. Extensive risk evaluations and clear regulatory channels will become necessary for large-scale adoption. The proposed approach will also integrate with climate-resilient bamboo management by addressing challenges associated with climate change through the adoption of sustainable practices.

Tri-kingdom biocontrol approaches offer a climate-resilient alternative to chemical control, by increasing eco-stability and functional redundancy. Beneficial microbial consortia act as a buffer against stress-induced vulnerability in bamboo, while viral regulators may potentially contribute to maintaining microbial population balance under changing environmental conditions, though direct evidence in bamboo is currently lacking (Wang X. et al., 2025; Wang Z. et al., 2025; Wang Y. et al., 2025). Table 3 summarizes the synergy mechanism and disruption strategies.

**Table 3:** Synergy mechanisms and corresponding disruption strategies for bamboo.

Synergy mechanism	Disruption strategy	Example (from other systems)
Infection court facilitation	Block fungal hyphal adhesion	Chitosan coating (rice)
Immunosuppression (BaMV)	Interfere with viral RNA silencing	Transgenic siRNA expression (model plants)
Metabolic cross-feeding	Scavenge iron via siderophores	Siderophore-producing *Bacillus* (maize)
Biofilm protection	Degrade biofilm matrix	Cellulase or DNase co-application (wheat)
Multiple mechanisms	Multi-strain consortia	*Trichoderma* + *Bacillus* (tomato)

Integration of tri-kingdom biocontrol strategies with sustainable management practices such as organic amendments, reduced chemical inputs, and precision irrigation can enhance disease suppression and plantation resilience (Farooq et al., 2023). However, practical implementation faces challenges related to scalability and economic feasibility. Advances in microbial mass production, formulation technologies, and delivery systems will be critical for large-scale adoption. In addition, standardized protocols for consortium design, quality control, and application timing are essential to ensure reproducibility and user confidence.

## 6 Challenges, knowledge gaps, and future perspectives

While significant progress has been achieved in understanding microbial behavior in relation to plant health, the application of tri-kingdom systems for the management of bamboo diseases remains at an early stage of development. The roles of fungi, bacteria, and viruses have been investigated independently in terms of disease suppression or pathogenicity; however, their combined effect in the bamboo microbiome remains poorly understood. Addressing these gaps is essential for translating mechanistic insights into durable and field-relevant disease management strategies and establishing bamboo as a paradigm system for sustainable perennial crop protection. Bamboo pathogens have not been studied using genome-wide association studies (GWAS), and no conserved genetic markers associated with synergistic virulence have been identified. Comparative genomics of synergistic and non-synergistic pathogen pairs should be used to identify candidate genes that mediate infection court and immunosuppression, as well as metabolic cross-feeding.

One of the most significant knowledge gaps is the scarcity of tri-kingdom studies in bamboo systems. Most current studies focus on individual microbial populations or pair-wise interactions, such as fungus-bacterium antagonism, while viral communities are frequently overlooked. From other plant systems, it has been clearly established that mycoviruses and bacteriophages play important roles in controlling the behaviors of microbial populations and pathogenicity, yet their roles in bamboo-associated microbial networks remains largely unexplored (Xie and Jiang, 2014). Viromic surveys combined with functional assays are therefore required to determine how viral agents influence fungal virulence, bacterial competitiveness, and stability of the microbiome in bamboo (Wang et al., 2018). Specifically: (i) no mycovirus has been isolated from a bamboo-infecting fungus, despite viromic evidence of their presence; (ii) no bacteriophage has been characterized against a bamboo bacterial pathogen; and (iii) the synergistic interactions of Bamboo mosaic virus (BaMV) with fungal or bacterial pathogens remain unquantified.

There are also methodological limitations that further constrain progress in this field. Most existing research relies on short-term greenhouse and laboratory experiments, which do not adequately capture the life cycle and ecological complexities of bamboo plantations. The perennial growth cycle, clonal reproduction, and high environmental plasticity of bamboo necessitate long-term, field-scale studies that incorporate temporal dynamics of bamboo microbiome (Fadiji et al., 2024). Moreover, although high-throughput sequencing has revealed extensive microbial diversity, functional validation of identified taxa remains limited. To move beyond the descriptive community profiles integrative multi-omics approaches including metagenomics, meta-transcriptomics, metabolomics and specialized gene expression analysis, are required to achieve a mechanistic understanding of tri-kingdom partnerships. The role of phylogenetic relatedness in mediating synergistic vs. antagonistic interactions remains unexplored in bamboo. Bacteria with similar hosts can be in more intense competition (competitive exclusion), whereas more distantly related species can be cooperative (niche complementarity). It remains unclear whether the synergy between *Fusarium* and *Erwinia* (S ≈ 1.8) is driven by phylogenetic distance or co-evolutionary history.

Another unsolved challenge concerns the stability and environmental compatibility of the microbial consortia. Artificially designed synthetic communities are often less stable when competing with native microbiota under environmental stresses. Soil heterogeneity, management strategies, and climatic variation strongly influence microbial colonization and interaction in bamboo systems. The design of context-adapted consortia that are compatible with local microbial communities and can maintain active interdependence over long periods should therefore be a primary focus of future research. Understanding how environmental factors and host-mediated selection shape microbiome assembly will be essential in enhancing predictability and sustainability of biocontrol interventions (Li et al., 2022).

Tri-kingdom disease management is further complicated by environmental and climatic factors. Climate change is anticipated to alter pathogen distribution, increase abiotic stress, and disrupt microbial balance within the bamboo plantation. Such changes may increase disease vulnerability and simultaneously reduce the effectiveness of traditional control strategies. Tri-kingdom approaches represent a promising strategy for climate-resilient disease management, yet their full potential cannot be realized without deeper insight into how microbial interactions respond to temperature extremes, drought, and nutrient limitation. Monitoring and modelling of microbiome dynamics under changing environmental conditions will be crucial for developing adaptive management approaches. To fully elucidate tri-kingdom synergy mechanisms in bamboo, integrated multi-omics approaches combining metagenomics, metabolomics, transcriptomics, and signaling molecules analysis are required. To date, no integrated multi-omics study has been conducted on bamboo, representing a critical methodological gap.

Integrating molecular interaction networks with practical disease management strategies should be a priority in future research. Understanding cross-kingdom communication will enable the rational design of targeted interventions by identifying the key signaling molecules, regulatory genes, and metabolic pathways. Recent advances in genome editing, functional genomics, and systems of biology have provided powerful tools for deconstructing these interactions and optimizing microbial mixtures to manage disease complexes. Notably, these advances must be coupled with intense biosafety studies and establishment of effective regulatory guidelines, especially with viral-based biocontrol agents, to guarantee environmental safety and public acceptance.

At a broader scale, tri-kingdom disease management strategies align with global efforts to reduce chemical inputs to enhance ecosystem services and promote sustainable agriculture and forestry. Bamboo plays a vital role in carbon sequestration, land restoration, and green economic growth, especially in the subtropical and tropical regions. Enhancing bamboo disease resistance through microbiome-based approaches will, therefore, extend beyond the crop protection, potentially contributing to environmental sustainability and climate change mitigation, though direct evidence for these broader impacts remains to be established.

In conclusion, despite the significant challenges, integrating fungi, bacteria, and viruses into a unified tri-kingdom framework represents a new paradigm in bamboo disease management. Mechanistic and multi-omics research will be necessary to fill existing knowledge gaps and will advance this field. Through the alignment of scientific innovation and sustainability objectives, future research can establish tri-kingdom interactions as a foundation for resilient and sustainable bamboo production.

## References

[ref1] Abdel-Rahman T. F. El-Morsy S. Halawa A. (2020). Occurrence of stem and leaf spots on lucky bamboo (*Dracaena sanderiana* hort. Ex. mast.) plants in vase and its cure with safe means. J. Plant Protect. Pathol. 11, 705–713. doi: 10.21608/jppp.2020.170648

[ref2] Ahmad Z. Kumari R. Mir B. Saeed T. Firdaus F. Vijayakanth V. . (2025). Bamboo for the future: from traditional use to industry 5.0 applications. Plants 14:3019. doi: 10.3390/plants14193019, 41095161 PMC12526147

[ref3] Alkhalil S. S. (2023). The role of bacteriophages in shaping bacterial composition and diversity in the human gut. Front. Microbiol. 14:1232413. doi: 10.3389/fmicb.2023.1232413, 37795308 PMC10546012

[ref4] Aoki N. Moriyama H. Kodama M. Arie T. Teraoka T. Fukuhara T. (2009). A novel mycovirus associated with four double-stranded RNAs affects host fungal growth in *Alternaria alternata*. Virus Res. 140, 179–187. doi: 10.1016/j.virusres.2008.12.003, 19118588

[ref5] Arasu A. (2025). Polymicrobial biofilms: biological insights and emerging directions in drug discovery. Future Microbiol. 20, 1323–1344. doi: 10.1080/17460913.2025.2604691, 41450110 PMC12928632

[ref6] Berrios L. Yeam J. Holm L. Robinson W. Pellitier P. T. Chin M. L. . (2023). Positive interactions between mycorrhizal fungi and bacteria are widespread and benefit plant growth. Curr. Biol. 33:2878-2887. e2874. doi: 10.1016/j.cub.2023.06.010, 37369208

[ref7] Bhardwaj P. Awasthi P. Prakash O. Sood A. Zaidi A. Hallan V. (2017). Molecular evidence of natural occurrence of apple stem grooving virus on bamboos. Trees 31, 367–375. doi: 10.1007/s00468-016-1375-8

[ref8] Bi L. Islam Z. F. Chan L. H. Hu H. W. (2025). The role of Phyllosphere microbes and viruses in biocontrol of pathogenic Fungi. Microb. Biotechnol. 18:e70251. doi: 10.1111/1751-7915.70251, 41074488 PMC12514008

[ref9] Borah R. K. (2006). Studies on the Incidence and Management of Culm Rot and Bamboo Blight Disease. Jorhat: Assam Agricultural University.

[ref10] Chauhan A. (2021). Studies on the Microbial Consortium Mediated Plant Defence for the Management of Damping-off of *Cedrus deodara* (Roxb.) G. Don Seedlings. Nauni, Solan, Himachal Pradesh, India: Dr Yashwant Singh Parmar University of Horticulture and Forestry.

[ref11] Cheng C.-P. (2017). Host factors involved in the intracellular movement of bamboo mosaic virus. Front. Microbiol. 8:759. doi: 10.3389/fmicb.2017.00759, 28487692 PMC5403954

[ref12] Cunniffe N. (2024). A synoptic review of plant disease epidemics and outbreaks published in 2022. Phytopathology. 114, 1717–1732. doi: 10.1094/PHYTO-01-24-0042-RVW 38723169

[ref13] Dey S. Biswas S. Kundu A. Pal A. Das M. (2023). “Current understanding on major bamboo diseases, pathogenicity, and resistance genes,” in Das, M., Ma, L., Pal, A., and Kole, C., Eds., Genetics, Genomics and Breeding of Bamboos, (Boca Raton, Florida, USA: CRC Press), 256–278.

[ref14] Dhuldhaj U. Pandya U. (2021). “Diversity, function, and application of fungal iron chelators (Siderophores) for integrated disease management,” in Seneviratne, G. and Zavahir, J. S., Eds., Role of Microbial Communities for Sustainability, (Singapore: Springer), 259–288.

[ref15] Dutta S. Gorain S. Roy J. Das R. Banerjee S. Gorai S. K. . (2025). Bamboo for global sustainability: a systematic review of its environmental and ecological implications, climate action, and biodiversity contributions. Environ. Rev. 33, 1–26. doi: 10.1139/er-2025-0032

[ref16] El-Ramady H. Abdalla N. Sári D. Ferroudj A. Muthu A. Prokisch J. . (2023). Nanofarming: promising solutions for the future of the global agricultural industry. Agronomy 13:1600. doi: 10.3390/agronomy13061600

[ref17] Elshahawy I. E. Darwesh O. M. (2023). Preventive and curative effect of difenoconazole+ azoxytrobin and thiophanate-methyl against lucky bamboo anthracnose disease caused by *Colletotrichum dracaenophilum*. Heliyon 9:e14444. doi: 10.1016/j.heliyon.2023.e14444, 36925537 PMC10011002

[ref18] Fadiji A. E. Adeniji A. Lanrewaju A. A. Adedayo A. A. Chukwuneme C. F. Nwachukwu B. C. . (2025). Key challenges in plant microbiome research in the next decade. Microorganisms 13:2546. doi: 10.3390/microorganisms13112546, 41304231 PMC12654229

[ref19] Fadiji A. E. Xiong C. Egidi E. Singh B. K. (2024). Formulation challenges associated with microbial biofertilizers in sustainable agriculture and paths forward. J. Sustain. Agric. Environ. 3:e70006. doi: 10.1002/sae2.70006

[ref20] Farooq M. Gogoi N. Pisante M. (2023). Sustainable Agriculture and the Environment. London, United Kingdom: Academic Press.

[ref21] Fishman J. A. (2013). Opportunistic infections—coming to the limits of immunosuppression? Cold Spring Harb. Perspect. Med. 3:a015669. doi: 10.1101/cshperspect.a015669, 24086067 PMC3784816

[ref22] García-Pedrajas M. Cañizares M. Sarmiento-Villamil J. L. Jacquat A. G. Dambolena J. S. (2019). Mycoviruses in biological control: from basic research to field implementation. Phytopathology 109, 1828–1839. doi: 10.1094/PHYTO-05-19-0166-RVW, 31398087

[ref23] Geng X.-S. Shu J.-P. Sheng J.-L. Zhang W. Peng H. (2020). Isolation and Identification of the Pathogens Causing Witches' Broom Disease of Five Bamboo Species of Non-Phyllostachys.

[ref24] Ghode N. (2023). Manual on Bamboo Cultivation: Bamboo Cultivation in Chhattisgarh, India: A Sustainable and Economically Viable Solution. Chhattisgarh, India: Shashwat Publication.

[ref25] Guerret M. G. Barbetti M. J. You M. P. Jones R. A. (2016). Effects of temperature on disease severity in plants of subterranean clover infected singly or in mixed infection with bean yellow mosaic virus and Kabatiella caulivora. J. Phytopathol. 164, 608–619. doi: 10.1111/jph.12484

[ref26] Guo H. Tan J. Jiao Y. Huang B. Ma R. Ramakrishnan M. . (2024). Genome-wide identification and expression analysis of the HAK/KUP/KT gene family in Moso bamboo. Front. Plant Sci. 15:1331710. doi: 10.3389/fpls.2024.1331710, 38595761 PMC11002169

[ref27] Hada S. Roat P. Chechani B. Kumar S. Yadav D. K. Kumari N. (2020). An overview on biomass of bamboo as a source of bioenergy. In N. Kumar Ed., Biotechnol. Biofuels, Singapore: Springer. 241–265. doi: 10.007/978-981-15-3761-5_10

[ref28] Hailemariam E. K. Hailemariam L. M. Amede E. A. Nuramo D. A. (2023). Identification of barriers, benefits and opportunities of using bamboo materials for structural purposes. Eng. Constr. Archit. Manag. 30, 2716–2738. doi: 10.1108/ECAM-11-2021-0996

[ref29] Hodiyah I. Benatar G. V. Sudartini T. Febryani N. Fitria A. D. Juhaeni A. H. (2024). Antagonistic potential of *Trichoderma* isolates from bamboo and cardamom rhizospheres against chili anthracnose pathogen. Int. J. Design Nat. Ecodynam. 19, 1847–1857. doi: 10.18280/ijdne.190602

[ref30] Huang Y.-P. Chen I.-H. Tsai C.-H. (2017). Host factors in the infection cycle of bamboo mosaic virus. Front. Microbiol. 8:437. doi: 10.3389/fmicb.2017.00437, 28360904 PMC5350103

[ref31] Huang Y.-W. Lee C.-W. Lin N.-S. Cuong H. V. Hu C.-C. Hsu Y.-H. (2022). First report of distinct bamboo mosaic virus (BaMV) isolates infecting *Bambusa funghomii* in Vietnam and the identification of a highly variable region in the BaMV genome. Viruses 14:698. doi: 10.3390/v14040698, 35458428 PMC9032891

[ref32] Hyde K. Zhou D. Dalisay T. (2002). Bambusicolous fungi: a review. Fungal Diversity. 9, 1–14.

[ref33] Jones P. Garcia B. J. Furches A. Tuskan G. A. Jacobson D. (2019). Plant host-associated mechanisms for microbial selection. Front. Plant Sci. 10:452782. doi: 10.3389/fpls.2019.00862, 31333701 PMC6618679

[ref34] Joshi T. Sharma P. Joshi T. Pandey S. C. Pande V. Pandey A. . (2020). A spotlight on the recent advances in bacterial plant diseases and their footprint on crop production. In recent advancements in microbial diversity (Academic Press). 37–69. doi: 10.1016/B978-0-12-821265-3.00003-7

[ref121] Kawadkar S. N. (2012). Survey and Collection of Disease Sample of Bamboo from Nagpur Vicinity (Thesis, Nagpur, India: Dendrocalamus strictus).

[ref35] Ke D. Luo J. Liu P. Shou L. Ijaz M. Ahmed T. . (2024). Advancements in bacteriophages for the fire blight pathogen *Erwinia amylovora*. Viruses 16:1619. doi: 10.3390/v16101619, 39459951 PMC11512310

[ref36] Kumar E. A. Rao I. R. Sastry C. (2021). Diseases and Disorders of Bamboos in Asia. Bamboo for Sustainable Development: Proceedings of the Vth International Bamboo Congress and the Vith International Bamboo Workshop, San José, Costa Rica, Leiden, Netherlands: VSP and INBAR (International Network for Bamboo and Rattan). 2–6.

[ref37] Lamichhane J. R. Venturi V. (2015). Synergisms between microbial pathogens in plant disease complexes: a growing trend. Front. Plant Sci. 6:385. doi: 10.3389/fpls.2015.00385, 26074945 PMC4445244

[ref38] Li Z.-Z. Luan Y. Hu J.-B. Fang C.-H. Liu L.-T. Ma Y.-F. . (2022). Bamboo heat treatments and their effects on bamboo properties. Constr. Build. Mater. 331:127320. doi: 10.1016/j.conbuildmat.2022.127320

[ref39] Li L. Wang Y. Yu C. Li S. Lin T. Han S. . (2023). Seasonal changes in the abundance *fusarium proliferatium*, microbial endophytes and nutrient levels in the roots of hybrid bamboo *Bambusa pervariabilis*× *Dendrocalamopsis grandis*. Front. Plant Sci. 14:1185449. doi: 10.3389/fpls.2023.1185449, 37538062 PMC10394707

[ref40] Lin T. Li L. Gu X. Owusu A. M. Li S. Han S. . (2023). Seasonal variations in the composition and diversity of rhizosphere soil microbiome of bamboo plants as infected by soil-borne pathogen and screening of associated antagonistic strains. Ind. Crop. Prod. 197:116641. doi: 10.1016/j.indcrop.2023.116641

[ref41] Liu L. Yang C. Liang F. Li C. Zeng Q. Han S. . (2024). Genome-wide survey of the bipartite structure and pathogenesis-related genes of *Neostagonosporella sichuanensis*, a causal agent of Fishscale bamboo rhombic-spot disease. Front. Microbiol. 15:1456993. doi: 10.3389/fmicb.2024.1456993, 39360322 PMC11444983

[ref42] MacAulay S. (2023). Reeling in the Truth: Drivers of Aquatic Infection and Control. Cardiff, United Kingdom: Cardiff University.

[ref43] Maina J. W. (2023). Adoption of Sustainable Bamboo Farming to Mitigate the Effects of Soil Degradation and to Improve Livelihood in Kinale. Kiambu County: Kenya Kenyatta University.

[ref44] Maliang H. Wang P. Chen A. Liu H. Lin H. Ma J. (2021). Bamboo tar as a novel fungicide: its chemical components, laboratory evaluation, and field efficacy against false smut and sheath blight of rice and powdery mildew and *fusarium* wilt of cucumber. Plant Dis., 105, 331–338. ddoi: 10.1094/PDIS-06-20-1157-RE, 32772833

[ref45] Mang S. M. Marcone C. Marcone M. Malvasi G. Chira D. Chira F. . (2025). Molecular characterization of *Cryphonectria parasitica* isolates from Basilicata region (southern Italy) and mycovirus identification. J. Biol. Res. 98, 12459. doi: 10.4081/jbr.2024.12459

[ref46] Mehrotra M. (1992). Diseases of plantation forestry in relation to paper industry and their management. IPPTA 4, 122–122.

[ref47] Meng M. Lee C.-C. (2017). Function and structural organization of the replication protein of bamboo mosaic virus. Front. Microbiol. 8:522. doi: 10.3389/fmicb.2017.00522, 28400766 PMC5368238

[ref50] Ning R. Li C. Xia M. Zhang Y. Gan Y. Huang Y. . (2024). *Pseudomonas*-associated bacteria play a key role in obtaining nutrition from bamboo for the giant panda (*Ailuropoda melanoleuca*). Microbiol. Spectrum 12, e0381923–e0303823. doi: 10.1128/spectrum.03819-23, 38305171 PMC10913395

[ref51] Nishad R. Ahmed T. Rahman V. J. Kareem A. (2020). Modulation of plant defense system in response to microbial interactions. Front. Microbiol. 11:1298. doi: 10.3389/fmicb.2020.01298, 32719660 PMC7350780

[ref52] Niu B. Wang W. Yuan Z. Sederoff R. R. Sederoff H. Chiang V. L. . (2020). Microbial interactions within multiple-strain biological control agents impact soil-borne plant disease. Front. Microbiol. 11:585404. doi: 10.3389/fmicb.2020.585404, 33162962 PMC7581727

[ref53] Nuss D. L. (2005). Hypovirulence: mycoviruses at the fungal–plant interface. Nat. Rev. Microbiol. 3, 632–642. doi: 10.1038/nrmicro1206, 16064055

[ref54] Pan C. Zhou G. Shrestha A. K. Chen J. Kozak R. Li N. . (2023). Bamboo as a nature-based solution (NbS) for climate change mitigation: biomass, products, and carbon credits. Climate 11:175. doi: 10.3390/cli11090175

[ref55] Ramsey M. M. Rumbaugh K. P. Whiteley M. (2011). Metabolite cross-feeding enhances virulence in a model polymicrobial infection. PLoS Pathog. 7:e1002012. doi: 10.1371/journal.ppat.1002012, 21483753 PMC3069116

[ref56] Ren B. Shi X. Guo J. Jin P. (2024). Novel insights on biofilm development in sewers: cross-kingdom exchange of quorum-sensing signaling molecules. J. Clean. Prod. 484:144302. doi: 10.1016/j.jclepro.2024.144302

[ref57] Rice S. A. Givskov M. Steinberg P. Kjelleberg S. (1999). Bacterial signals and antagonists: the interaction between bacteria and higher organisms. J. Mol. Microbiol. Biotechnol. 1, 23–31, 10941781

[ref58] Santra H. K. Banerjee D. (2020). “Fungal endophytes: a source for biological control agents,” in Yadav, A.N., Mishra, S., Kour, D., Yadav, N., and Kumar, A., Eds., Agriculturally Important Fungi for Sustainable Agriculture: Volume 2: Functional Annotation for Crop Protection, (Singapore: Springer), 181–216.

[ref59] Sapovadia V. K. (2025). Unlocking Bamboo's potential: diversifying products and driving sustainable development through innovation. doi: 10.2139/ssrn.5285960

[ref60] Sharma N. Pal J. Mahajan R. Chandel S. Sud D. Sanspal A. (2026). Pathogens without borders: a review on cross-kingdom transmission strategies and pathogenicity of plant and human pathogens. Arch. Microbiol. 208:98. doi: 10.1007/s00203-025-04633-4, 41493483

[ref61] Shukla A. Singh A. Tiwari D. Ahirwar B. K. (2016). Bambusicolous fungi: a reviewed documentation. Int. J. Pure Appl. Biosci 4, 304–310. doi: 10.18782/2320-7051.2268

[ref62] Siddique A. B. (2020). Viruses of endophytic and pathogenic forest fungi. Virus Genes 56, 407–416. doi: 10.1007/s11262-020-01763-3, 32388614 PMC7329786

[ref63] Sriragavi G. Sangeetha M. Santhakumar M. Lokesh E. Nithyalakshmi M. Saleel C. A. . (2023). Exploring antibacterial properties of bioactive compounds isolated from *Streptomyces* sp in bamboo rhizosphere soil. ACS Omega 8, 36333–36343. doi: 10.1021/acsomega.3c04954, 37810705 PMC10552487

[ref64] Tayi L. (2025). Players in action: role of metabolites in plant defence. Advanc. Biochem. Molec. Mechan. Plant Pathogen Interact. 81:819. doi: 10.1088/978-0-7503-5673-2ch8

[ref65] Thiribhuvanamala G. Parthiban K. Seenivasan R. (2017). Major diseases affecting pulp wood trees and their management. Plantation and Agroforestry: Pulpwood Value Chain Approach, Scientific Publishers, 337. doi: 10.1088/978-0-7503-5673-2ch8, 30793291

[ref66] Tian X.-K. Wang M.-Y. Meng P. Zhang J.-S. Zhou B.-Z. Ge X.-G. . (2020). Native bamboo invasions into subtropical forests alter microbial communities in litter and soil. Forests 11:314. doi: 10.3390/f11030314

[ref67] Wagemans J. Holtappels D. Vainio E. Rabiey M. Marzachì C. Herrero S. . (2022). Going viral: virus-based biological control agents for plant protection. Annu. Rev. Phytopathol. 60, 21–42. doi: 10.1146/annurev-phyto-021621-114208, 35300520

[ref68] Wang X. Li Y. Kang L. Zhang Z. Zhang D. Li P. . (2025). Diversity, functions, and antibiotic resistance genes of bacteria and fungi are examined in the bamboo plant phyllosphere that serve as food for the giant pandas. Int. Microbiol. 28, 751–763. doi: 10.1007/s10123-024-00583-x, 39168909 PMC11991987

[ref69] Wang Z. Li J. Shi M. Qing H. Zhang Y. Jing R. . (2025). Microbes-mediated adaptive mechanisms support asymmetric drought resistance of clonal ramets for Moso bamboo. Ind. Crop. Prod. 236:121971. doi: 10.1016/j.indcrop.2025.121971

[ref70] Wang Y. Ren H. Zhong Y. Song R. Jiang S. Lai M. . (2025). Microbial diversity and function in bamboo ecosystems. Front. Microbiol. 16:1533061. doi: 10.3389/fmicb.2025.1533061, 40636498 PMC12238013

[ref71] Wang H. Wang X. Cui Y. Xue Z. Ba Y. (2018). Slow pyrolysis polygeneration of bamboo (*Phyllostachys pubescens*): product yield prediction and biochar formation mechanism. Bioresour. Technol. 263, 444–449. doi: 10.1016/j.biortech.2018.05.040, 29772506

[ref72] Wang X. Wang S. Huang M. He Y. Guo S. Yang K. . (2024). Phages enhance both phytopathogen density control and rhizosphere microbiome suppressiveness. MBio 15, e0301623–e0303023. doi: 10.1128/mbio.03016-23, 38780276 PMC11237578

[ref73] Wang K. Wu Y. Ye M. Yang Y. Asiegbu F. O. Overmyer K. . (2021). Comparative genomics reveals potential mechanisms of plant beneficial effects of a novel bamboo-endophytic bacterial isolate *Paraburkholderia sacchari* Suichang626. Front. Microbiol. 12:686998. doi: 10.3389/fmicb.2021.686998, 34220778 PMC8250432

[ref74] Wongsuk T. Pumeesat P. Luplertlop N. (2016). Fungal quorum sensing molecules: role in fungal morphogenesis and pathogenicity. J. Basic Microbiol. 56, 440–447. doi: 10.1002/jobm.201500759, 26972663

[ref75] Wu D. Wang W. Yao Y. Li H. Wang Q. Niu B. (2023). Microbial interactions within beneficial consortia promote soil health. Sci. Total Environ. 900:165801. doi: 10.1016/j.scitotenv.2023.165801, 37499809

[ref116] Wu L. Yang J. Gu Y. Wang Q. Zhang Z. Guo H. . (2025). Bamboo mosaic virus‐mediated transgene‐free genome editing in bamboo. New Phytologist, 245, 1810–1816. 39763115 10.1111/nph.20386

[ref76] Wu Q. Zhang L. Xia H. Yu C. Dou K. Li Y. . (2017). Omics for understanding synergistic action of validamycin a and *Trichoderma asperellum* GDFS1009 against maize sheath blight pathogen. Sci. Rep. 7:40140. doi: 10.1038/srep40140, 28057927 PMC5216365

[ref77] Xie J. Jiang D. (2014). New insights into mycoviruses and exploration for the biological control of crop fungal diseases. Annu. Rev. Phytopathol. 52, 45–68. doi: 10.1146/annurev-phyto-102313-050222, 25001452

[ref78] Xie J. Jiang D. (2024). Understanding the diversity, evolution, ecology, and applications of mycoviruses. Ann. Rev. Microbiol. 78, 595–620. doi: 10.1146/annurev-micro-041522-105358, 39348839

[ref79] Yu S.-S. Wei W. (2025). Beyond single-pathogen models: understanding mixed infections involving Phytoplasmas and other plant pathogens. Plants 14:2049. doi: 10.3390/plants14132049, 40648058 PMC12251814

[ref80] Yuan Z.-S. Liu F. Zhang G.-F. (2015). Characteristics and biodiversity of endophytic phosphorus-and potassium-solubilizing bacteria in Moso bamboo (*Phyllostachys edulis*). Acta Biol. Hung. 66, 449–459. doi: 10.1556/018.66.2015.4.9, 26616376

[ref81] Zhang L. (2023). Bamboo Expansion: Processes, Impacts, and Management. Singapore: Spring.

[ref82] Zhang Z. Cao Y. Sun M. Wang L. Yang H. (2025). Phenological shifts drive rhizosphere microbial community dynamics in subtropical woody bamboo (*Chimonobambusa utilis* (Keng) PC Keng): pH and total phosphorus as main drivers. Rhizosphere 34:101072. doi: 10.1016/j.rhisph.2025.101072

[ref83] Zhang X. Gao G. Wu Z. Wen X. Zhong H. Zhong Z. . (2019). Agroforestry alters the rhizosphere soil bacterial and fungal communities of moso bamboo plantations in subtropical China. Appl. Soil Ecol. 143, 192–200. doi: 10.1016/j.apsoil.2019.07.019

[ref84] Zhang J. Ning Y. Wu H. Gao G. Wu Z. Peng Y. . (2024). Intercropping shapes the metabolome and microbiome of medicinal Giant lily (*Cardiocrinum giganteum*) in bamboo, Chinese fir, and mixed forests. Forests 15:2201. doi: 10.3390/f15122201

[ref85] Zhang M. Zhou Y. Cui X. Zhu L. (2024). The potential of co-evolution and interactions of gut bacteria–phages in bamboo-eating pandas: insights from dietary preference-based metagenomic analysis. Microorganisms 12:713. doi: 10.3390/microorganisms12040713, 38674657 PMC11051890

[ref86] Zhao B. Deng J. Liu R. Xu G. Cao Y. Hu S. (2025). Construction of a co-culture consortium for the effective degradation of bamboo lignin and its potential application in seedling substrate. Ind. Crop. Prod. 225:120589. doi: 10.1016/j.indcrop.2025.120589

[ref87] Zhu Q. Shi N. Wang P. Zhang Y. Peng F. Yang G. . (2022). A novel gammapartitivirus that causes changes in fungal development and multi-stress tolerance to important medicinal fungus *Cordyceps chanhua*. J. Fungi 8:1309. doi: 10.3390/jof8121309, 36547642 PMC9782574

